# Morphology, Biochemical, and Molecular Characterization of *Pasteurella multocida* Causing Hemorrhagic Septicemia in Indonesia

**DOI:** 10.1155/2023/7778707

**Published:** 2023-03-11

**Authors:** Muhammad Ibrahim Desem, Ekowati Handharyani, Agus Setiyono, Safika Safika, Didik Tulus Subekti, Fitrine Ekawasti

**Affiliations:** ^1^National Research and Innovation Agency, Jakarta Pusat 10340, Indonesia; ^2^Graduate School of Veterinary Medicine and Biomedicine, Bogor Agricultural University, Bogor 16880, Indonesia; ^3^Department of Clinic, Reproduction and Pathology, School of Veterinary Medicine and Biomedicine, Bogor Agricultural University, Bogor 16880, Indonesia; ^4^Department of Animal Disease and Animal Public Health, School of Veterinary Medicine and Biomedicine, Bogor Agricultural University, Bogor 16880, Indonesia; ^5^Center for Biomedical Research, National Research and Innovation Agency, Bogor 16911, Indonesia

## Abstract

*Pasteurella multocida* is a Gram-negative bacterium that causes hemorrhagic septicemia (HS) in buffaloes and cattle. The disease causes serious problems in Indonesian livestock and is classified as a serious transmissible animal disease. Previous research has determined the diversity of *P. multocida* using a serotyping method based on the antigenic properties of capsule polysaccharides. An alternative method for analysis utilizes sodium dodecyl sulfate-polyacrylamide gel electrophoresis (SDS-PAGE) and random amplified polymorphic DNA (RAPD). This study aimed to characterize and determine *P. multocida* diversity in several regions of Indonesia based on phenotypic character, protein profile, and the band pattern of RAPD results. Bacterial identification was performed using traditional biochemical techniques and API® 20NE systems and then confirmed molecularly using polymerase chain reaction (PCR). The freeze-thawing technique was performed to obtain the bacterial protein extract, and DNA extraction was executed using DNAzol. The extracted protein and RAPD product were then electrophoresed on 12% polyacrylamide gel and 1.5% agarose gel, respectively. The results indicate that the molecular weight range of the protein bands is 12–209 kDa, and the band pattern of the RAPD results ranged from 307–3,100 bp. Based on phenotypical analysis, *P. multocida* from South Sulawesi Province exhibited a variety of growth characteristics in MacConkey agar media. Using the hierarchical clustering analysis of the band patterns of RAPD and the whole-cell protein profiles, four and five clusters were formed, respectively. These results indicate molecular diversity among *P. multocida* from several regions of Indonesia.

## 1. Introduction

*Pasteurella multocida* is an aerobic, Gram-negative bacteria with a short rod or coccoid structure. It is approximately 0.2–0.4 × 0.6–2.5 *µ*m in size and is classified as encapsulated (usually in virulent strains), nonmotile, and nonspore-forming. Furthermore, it exhibits a bipolar appearance in Gram staining. The most significant biochemical characteristics for identification include indole production, nonhemolysis against sheep blood, positive oxidase reactions, lack of growth on MacConkey agar, and fermenting hexose sugar groups [1]. In ruminant species, *P. multocida* causes hemorrhagic septicemia (HS), whereas in avian species, it causes fowl cholera, and in swine, it causes atrophic rhinitis [2]. Between 2005 and 2019, there were 105,692,984 instances of HS reported across 41 nations. The total number of deaths was 99,550, accounting for 0.1% of all cases and costing USD 692,092.90 yearly [3]. In Indonesia, HS is classified as a serious transmissible animal disease by the Ministerial Decree of the Minister of Agriculture of the Republic of Indonesia, number 4026/KPTS/OT.140/4/2013.

The diversity of *P. multocida* is commonly detected through serotyping. The serotyping classification is based on the antigenic properties of the capsule polysaccharide and the lipopolysaccharide (LPS) composition. A passive hemagglutination test has been used to classify *P. multocida* into five serogroups (A, B, D, E, and F) based on the polysaccharide capsule structure, and a gel diffusion precipitation test has classified it into 16 serotypes (1–16) based on the composition of LPS [4–8]. However, this method has the disadvantage of being time-consuming and requiring specific antisera. These specific antisera are difficult to produce, especially the antisera against capsule polysaccharides, because they are less immunogenic [2, 8]. The alternatives for biodiversity analysis include the use of sodium dodecyl sulfate-polyacrylamide gel electrophoresis (SDS-PAGE) and random amplified polymorphic DNA (RAPD). This method has been demonstrated using various species of microorganisms. Previous studies have shown that SDS-PAGE can be used to conduct biodiversity analysis based on bacterial protein profiles [9–12]. Therefore, the diversity of *P. multocida* could also be detected using RAPD [13–16].

It is necessary to analyze the diversity of *P. multocida* because research has long established that Indonesia possesses a rich biodiversity of microorganisms [17]. Therefore, the diversity of *P. multocida* from Indonesia is expected to be high. To the best of our knowledge, there has been no previous research on the diversity of *P. multocida* from Indonesia based on RAPD patterns and whole-cell protein profiles. This characterization is important because it can serve as a cornerstone for studying the diversity of *P. multocida* from Indonesia and predicting its virulence profile. Furthermore, these findings can be utilized in developing an immunodiagnostic kit because protein, one of the antigenic components of *P. multocida* [2, 18–21], is commonly used in the development of immunodiagnostic kits.

## 2. Materials and Methods

### 2.1. Cultural and Biochemical Identification of *P. multocida*

Ten *P. multocida* isolates from the Indonesian Research Center for Veterinary Science's collection were used in this study, consisting of nine isolates from several regions in Indonesia and one standard isolate of *P. multocida* B:2 (Table 1). These isolates were cultured on 5% sheep blood agar (SBA) (Oxoid Ltd., UK) and MacConkey agar (Oxoid Ltd., UK), followed by incubation at 37°C for 24 h. The plates were then examined for growth. Next, the colonies were Gram stained and tested, then observed for the following characteristics: no growth on MacConkey agar, catalase and oxidase positivity, indole production, nonmotility, nonhemolysis on SBA, and acid from glucose [22, 23]. Biochemical identification was also performed using an API® 20NE kit (bioMérieux, France) based on its instruction manual.

### 2.2. DNA Extraction

Genomic DNA was extracted using DNAzol (Molecular Research Center, Inc., USA) and preserved at −20°C until further analysis.

### 2.3. Identification of *P. multocida* Based on *23S rRNA* and *ompH* Genes

The *23S rRNA* and *ompH* genes were amplified in PCR using oligonucleotide primers, using the methods previously described by Miflin and Blackall [24] and Luo et al. [25], respectively (Table 2). PCR was performed using 20 ng of genomic DNA along with forward and reverse primers (10 pmol each) in a MyTaq HS Red Mix (Bioline, USA) master mix. The PCR-amplified product was analyzed on a 1% agarose gel along with a DNA molecular weight marker.

### 2.4. RAPD

The RAPD amplification was performed using M13 primer based on Taylor et al.'s method [26] (Table 2), using 20 ng of genomic DNA and 10 pmol of primer in an *i*-Taq master mix solution (Intron, Republic of Korea). The RAPD product was analyzed on a 1.5% agarose gel along with a DNA molecular weight marker.

### 2.5. Protein Extraction

Bacterial whole-cell protein was extracted based on the modified method described by Rachmawati et al. [27]. In brief, bacterial cells were washed twice with phosphate-buffered saline by centrifugation at 7,500 × *g* for 15 minutes (M-Science, MPW Med. Instruments, Poland). The soluble protein was obtained by freeze-thawing the bacterial suspension in liquid nitrogen, and the suspension was centrifuged at 920 × *g* for 10 minutes. The supernatant was then collected and preserved at −20°C until further analysis.

### 2.6. Protein Electrophoresis (SDS-PAGE)

Electrophoresis was performed on a 12% polyacrylamide gel (Invitrogen, USA) using the method by Laemmli [28]. In short, 10 µg protein and a Laemmli sample buffer (Bio-Rad, USA) were homogenized at a 1 : 1 ratio. Protein samples, along with a protein molecular weight marker (Thermo Scientific, Lithuania), were electrophoresed at a constant voltage of 100 V. The protein bands were then stained using 0.1% Coomassie brilliant blue (AppliChem GmbH, Germany).

### 2.7. Data Analysis

Protein bands and RAPD patterns were analyzed using PyElph software version 1.4 to obtain binary matrix data. Hierarchical clustering analysis was performed using the Ward's linkage method on Minitab version 18 software.

## 3. Results

### 3.1. Bacterial Identification

**Figure 1:**
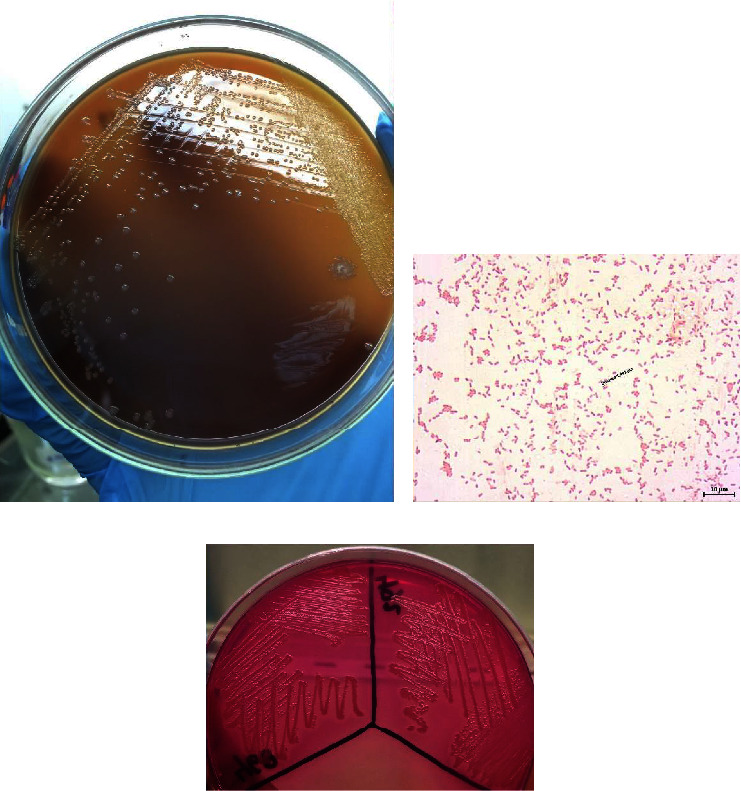
*P. multocida* colonies growing on sheep blood agar (a) and their characteristics on Gram staining at 1,000x magnification (b). Colonies of *P. multocida* isolates growing on MacConkey agar (c).

**Figure 2:**
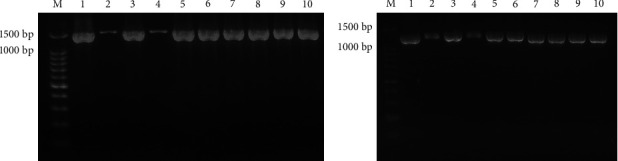
Results of PCR amplification targeting the *23S rRNA* (a) and *ompH* (b) genes in *P. multocida*. The *23S rRNA* gene is one of the target genes that can be used to identify *P. multocida*. Its amplification product size is approximately 1,400 bp [24]. The *ompH* gene is a virulent gene that belongs to *P. multocida,* with an amplification product size of approximately 1,000 bp [25]. M: DNA molecular markers; 1: BLT-18; 2: 492; 3: 471; 4: 468; 5: B2955; 6: B2951; 7: B2954; 8: B2953; 9: M1404; 10: NTT-16.

All isolates exhibited growth on SBA, and nonhemolysis was observed (Figure 1(a)). The bacterial cells' characteristics were Gram-negative with a coccoid bipolar structure (Figure 1(b)). All isolates were also observed to be nonmotile, catalase- and oxidase-positive, and indole positive, and they were all capable of producing acid from glucose. Eight isolates exhibited no growth on MacConkey agar, and they were correctly identified as *P. multocida* by API® 20NE with a numerical profile of 3000004 (% ID = 96%). This numerical profile is consistent with the results of previous studies [29, 30]. Meanwhile, the other two isolates (468 and 492) grew pink bacterial colonies on MacConkey agar (Figure 1(c)). Interestingly, the results of API® 20NE identified these two isolates as *Vibrio parahaemolyticus,* with a numerical profile of 7067746 (% ID = 98.9%), and *Aeromonas hydrophila,* with a numerical profile of 7467746 (% ID = 87%), respectively. However, molecular identification by PCR demonstrated that all the isolates used were *P. multocida* (Figures 2(a) and 2(b)).

### 3.2. *P. multocida* Diversity Based on RAPD

**Figure 3:**
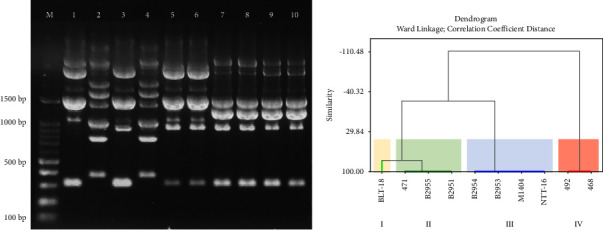
Variation of DNA fragment patterns of *P. multocida* isolates used in the study (a) and dendrogram generated using the Ward's linkage method (b). M: DNA molecular markers: 1: BLT-18; 2: 492; 3: 471; 4: 468; 5: B2955; 6: B2951; 7: B2954; 8: B2953; 9: M1404; 10: NTT-16.

RAPD showed variations in the pattern of DNA fragments against the 10 isolates of *P. multocida* used in this study. There were 6–8 bands generated in these fragment patterns, with a range ranging from 307–3,100 bp (Figure 3(a)). Hierarchical clustering analysis of DNA fragment patterns indicated the formation of four clusters, using a 95% similarity value (Figure 3(b)). These results are in line with the results of a study conducted by Chitarra et al. [31], who produced 7–11 bands and varied patterns of DNA fragments using the M13 primer. Of the four clusters formed in the present study, cluster III was the most frequently formed cluster (40%), followed by clusters II (30%), IV (20%), and I (10%). Strangely, isolates 468 and 492, which have the property of being able to grow on MacConkey agar, were grouped in separate clusters. These results indicate that genetic differentiation can be established between these two isolates when compared with other isolates that do not grow on MacConkey agar. These results support findings from phenotypical bacterial identification that demonstrate different growth characteristics on MacConkey agar.

### 3.3. *P. multocida* Diversity Based on Protein Profile

**Figure 4:**
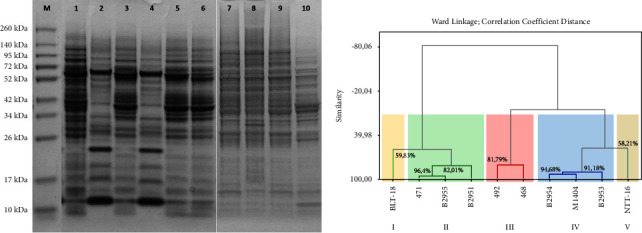
Whole-cell protein profiles of *P. multocida* isolates used in the study (a) and dendrogram generated using the Ward's linkage method (b). M: protein molecular weight markers: 1: BLT-18; 2: 492; 3: 471; 4: 468; 5: B2955; 6: B2951; 7: B2954; 8: B2953; 9: M1404; 10: NTT-16.

SDS-PAGE results indicated that the whole-cell protein band range of *P. multocida* isolates used in this study ranged from 12–209 kDa (Figure 4(a)). Hierarchical clustering analysis of protein profiles grouped 10 isolates into five clusters with a similarity value of 70% (Figure 4(b)). These results are consistent with previous studies: protein profile dendrograms with similarity values above 65% in *P. multocida* [32] and 70% in coagulase-negative staphylococci are considered sufficiently high for observing intraspecies diversity [11]. In this clustering, the isolates that were able to grow on MacConkey agar were grouped into one cluster (cluster III), and the other eight isolates were divided into four additional clusters. Four isolates from Lampung Province, namely, B2951, B2953, B2954, and B2955, were separated into two different clusters. In general, clustering based on protein profiles by SDS-PAGE and DNA fingerprinting by RAPD shows similar results.

## 4. Discussion

*P. multocida* exhibits a lack of growth on MacConkey agar. However, on rare occasions, it is still possible for several strains to grow on MacConkey agar. This evidence was reported by Heddleston and Wessman in a previous study [33], which found that one *P. multocida* isolate could grow on MacConkey agar out of a total of 1,088 isolates tested. Pink colonies also indicate that these bacteria exhibit the property of being able to ferment lactose. This characteristic is rarely found in *P. multocida*, but it can occasionally occur [34], as confirmed by the results of previous studies [33–37]. Cases of lactose fermentation in *P. multocida* generally occur in *P. multocida* biovars 12 and 14 [36].

Cases of misidentification in API® 20NE are most likely caused by the indicators used in its identification system. Such indicators are mostly derived from information concerning the ability of bacteria to utilize carbohydrates in their biological systems. If two bacterial species have similar carbohydrate utilization characteristics, API® 20NE will most likely misidentify the two species as the same species. This error will be problematic if this kit is used exclusively for identification, especially if the results provide a high identification percentage value, as in the results of this study. As in previous studies, the identification results of the API® kit were deemed accurate in this study if the identification percentage value exceeded 80% [38].

The biochemical characteristics of *Aeromonas* bacteria—especially those related to carbohydrate utilization—are similar to *P. multocida* [39]. The characteristics that can be used to distinguish *Pasteurella* from *Aeromonas* and *Vibrio* are their motility and type of hemolysis. *Aeromonas hydrophila* and *Vibrio parahaemolyticus* are motile bacteria [40, 41]. *Aeromonas* bacteria generally have the *β*-hemolysis type, while *P. multocida* have the nonhemolysis type [39]. Both of these characteristics are not accommodated by the API® 20NE test, so they can cause misidentification if not tested separately. Cases of misidentification by API® 20NE on *P. multocida* have also been reported in a previous study [30]. The misidentified isolate was identified as *Aeromonas salmonicida* (% ID = 55.2%). The study also found *P. multocida* isolates that were misidentified as *Vibrio hollisae* (% ID = 80.6%) using API® 20E. Other studies indicate that the API® identification system is not infallible, but its effectiveness still exceeds 80% [42–44]. Cumulatively, these findings support previous reports that the API® 20NE kit yields inconsistent identification percentages for the same organism. As such, the API® 20NE kit should be used to complement traditional identification systems, thereby minimizing the risk of misidentification.

The RAPD clustering performed in this study also supports the results of previous studies [26, 31, 45]. The present study's method demonstrates that the M13 primer can be used for genetic differentiation (DNA fingerprinting) of *P. multocida* and can thus describe the diversity among *P. multocida* isolates from Indonesia. The dendrogram also indicates the diversity among *P. multocida* isolates from the same region. Isolates from Lampung Province and South Sulawesi Province were grouped into two different clusters (clusters II and III for Lampung Province; clusters II and IV for South Sulawesi Province). Previous research in Brazil demonstrated that 14 *P. multocida* isolates sourced from the city of Diamantino could be grouped into two different clusters when analyzed using primer M13 [31]. Findings in other countries also indicate that *P. multocida* originating from one region exhibits high diversity when analyzed using RAPD. In India, *P. multocida* isolated from the Tamil Nadu region exhibited diversity that could be grouped into nine clusters [46]. Similar findings were also reported in the Republic of Korea, where *P. multocida* isolated from Gyeongsangbuk-do Province exhibited diversity that could be grouped into eight clusters [45]. These results indicate that RAPD is a simple and fast method for identifying patterns that are useful for strain differentiation [46].

The dendrogram based on SDS-PAGE also demonstrates the diversity of *P. multocida* from several regions in Indonesia. In cluster IV, isolates B2953 and B2954 were joined in one cluster with M1404. In this cluster, B2954 and M1404 exhibited the highest similarity level (94.68%). Meanwhile, isolates B2951 and B2955 were grouped in one cluster with isolate 471 (cluster II), which originated from South Sulawesi Province; these had a similarity level of 82.01%. Two other isolates, BLT-18 and NTT-16, which were isolated from dead cows with clinical symptoms of HS from two different provinces, formed a separate cluster. These findings indicate that, apart from reflecting the phenotypic character [32], the clusters can also be assumed to reflect the isolates' regions of origin. Furthermore, the diversity of protein profiles has consequences for the diversity of immunogenic proteins. These proteins can trigger antibody responses and be detected by a Western blot test [47].

The identification of isolates using the phenotypic, biochemical, and physiological characteristics of microorganisms is frequently impractical and inefficient, and the results are sometimes unreliable due to variations in the reactions from one test to the next [10, 11]. The classification method used for *P. multocida* serotypes is usually performed using time-consuming serological methods [4, 5], and the specific antisera are difficult to produce. SDS-PAGE is advantageous because it can be completed in one day, unlike conventional phenotypic methods. Information about the estimated genome of a microorganism can also be derived simply and quickly using a whole-cell protein profile. Whole-cell protein profiles also correlate with the biochemical characteristics of a species because different physiological characteristics tend to produce different protein profiles [10]. The results of this study support this assertion by illuminating the striking differences in RAPD and protein profiles between isolates that can grow on MacConkey agar and those that cannot.

The diversity of isolates from the same region is also observable based on their protein profiles. Isolates from Lampung Province and South Sulawesi Province were grouped into two clusters. Similar to the RAPD results, this finding indicates that *P. multocida* isolates from the same region are likely to be diverse. Similar findings have also been reported in a previous study [48], in which several *P. multocida* isolates from the same region had different protein profiles. For another example, isolates of *Trypanosoma evansi* from the same village in South Kalimantan Province had diverse protein profiles [49].

This study's findings can serve as an initial guideline for the development of an immunodiagnostic kit. As previously reported [47], the selection of isolates based on protein profiles and the characteristics of immunogen proteins as antigen candidates constitute important steps in the development of an enzyme-linked immunosorbent assay. Subekti and Yuniarto [47] reported that isolates with different protein profiles may yield a different image of specific immunogenic proteins when tested against the same standard sera. These isolates may even exhibit different nonspecific reactions or cross-reactions to the same negative standard serum. The same phenomenon has also been reported in other studies [21, 50]. These findings indicate that not all isolates are suitable for use as antigens in the development of an immunodiagnostic kit. The existence of diversity among *P. multocida* isolates from various regions of Indonesia is important initial information to guide the development of an immunodiagnostic kit and allow for further exploration of these isolates. Genome sequencing is recommended for analysis of sequence-based similarities. In addition, analysis of immunogenic proteins using a Western blot test also needs to be carried out at a later stage in the development of an immunodiagnostic kit.

## 5. Conclusion

In conclusion, the molecular weight range of the protein bands of *P. multocida* from several regions in Indonesia is 12–209 kDa, and the band pattern of the RAPD results ranged from 307–3,100 bp. Using the hierarchical clustering analysis of the band patterns of RAPD and the whole-cell protein profiles, four and five clusters were formed, respectively. These results indicate molecular diversity among *P. multocida* from several regions of Indonesia.

## Figures and Tables

**Table 1:** *P. multocida* isolates used in this study.

No.	Isolate code	Origin	Host
1	NTT-16	East Nusa Tenggara Province	Cattle
2	BLT-18	East Java Province	Cattle
3	B2951	Lampung Province	Cattle
4	B2953	Lampung Province	Cattle
5	B2954	Lampung Province	Cattle
6	B2955	Lampung Province	Cattle
7	468	South Sulawesi Province	Cattle
8	471	South Sulawesi Province	Unknown
9	492	South Sulawesi Province	Cattle
10	M1404	Ames, Iowa	Bison

**Table 2:** PCR primer sets used in this study.

Primer name	Sequence (5′ to 3′)	PCR program	Reference
PM23F1	GGCTGGGAAGCCAAATCAAAG	98°C 2.5 min, 30 × (94°C 1 min, 50°C 1 min, and 72°C 1 min), 72°C 10 min	[24]
PM23R2	CGAGGGACTACAATTACTGTAA		
ompH-F	ACTATGAAAAAGACAATCGTAG	94°C 5 min, 35 × (94°C 15 s, 55°C 1 min, and 72°C 1 min), 72°C 10 min	[25]
ompH-R	GATCCATTCCTTGCAACATATT		
M13	GAGGGTGGCGGTTCT	95°C 3 min, 30 × (95°C 30 s, 44°C 58 s, and 72°C 70 s), 72°C 7 min	[26]

## Data Availability

The data used to support the findings of this study are available from the corresponding author upon request.
